# Supplementary material for: Calorie and nutrient trends in large U.S. chain restaurants, 2012-2018
Source: PLoS One. 2020 Feb 10;15(2):e0228891. doi: 10.1371/journal.pone.0228891 (PMC7010289; doi:10.1371/journal.pone.0228891)
Supplement: S1 File — (DOCX) [file pone.0228891.s001.docx]

**S1 File. Covariate definitions**

**Restaurant-level covariates**

**Restaurant saturation**: For each restaurant, we used Google maps to code whether or not a restaurant had any location in each of the 9 U.S. Census Divisions. To indicate a national restaurant chain, a binary measure of whether a restaurant chain had locations in all 9 Census Divisions or not was created. This binary measure was included in the analysis after careful consideration of continuous measures of Census divisions (i.e., 1-9) or dummy variables for whether or not a restaurant had a location in each of the 9 Census divisions.^1^

**Restaurant type:** Each restaurant was coded as either full service, fast casual, or fast food. The first step was to look at restaurants’ websites to determine if they self-identified as full-service, fast causal, or fast-food/quick-service. If so, the restaurant’s self-identification was used. If not, full-service restaurants were defined as those with table service. Fast casual restaurants were defined as those that had at least two of the following four criteria: non-disposable utensils, onsite food preparation, no table service, and commitment to higher-quality or fresh ingredients or sustainability. Fast food restaurants were defined as those with no table service and meeting fewer than two of the above criteria.^2^

**Year that restaurant introduced menu labeling:**Restaurants were categorized as being an early adopter of menu labeling if they were identified in previous publications as having introduced labeling prior to 2014.^3^ Restaurants were categorized as being in early compliance with menu labeling if they were identified in previous publications as having introduced labeling prior to 2017, but not before the start of the study period.^4^ Restaurants were identified as being compliant with labeling if they introduced labeling in 2018, but not earlier, based on a further assessment from members of our research team (information not yet published); and restaurants were identified as non-compliant with labeling if they had not introduced menu labeling in 2018 in accordance with Affordable Care Act.^5^

**Item-level covariates**

**Children’s item:** The MenuStat data contained character variables with text describing menu item names and menu item descriptions. Any menu item or menu item description containing the words “kid”, “kids”, “child”, or “children” was classified to be a children’s menu item. This was a binary measure of whether or not an item was a children’s menu item.^1,6,7^

**Regionally offered:** Items were coded as regional by MenuStat if the restaurant describes the item as regional (e.g. Midwest states only or at participating locations only).^8^

**Limited time item:** Items were coded as “limited time” by MenuStat if the restaurant describes the item as a limited time offer or seasonal.^8^

**Shareable**: Items were coded as Shareable by MenuStat if the restaurant describes the item as shareable and the nutrition cannot be divided in to a single serving (e.g. carafes, whole pies, quarts of ice cream, 2 liter drinks). ^8^ For sensitivity analysis only, we also classify items as shareable that were not identified by the MenuStat team but had >2000 calories or >1000 calories for appetizers & sides.

**Other definitions**

**Menu Category:** MenuStat data are assigned to one of 12 mutually exclusive categories by the MenuStat team: (1) Appetizers & Sides, (2) Baked Goods, (3) Beverages, (4) Burgers, (5) Desserts, (6) Entrees, (7) Fried Potatoes, (8) Pizza, (9) Salads, (10) Sandwiches, (11) Soup, and (12) Toppings and Ingredients. ^8^ For the purpose of this analysis, we collapsed Baked Goods and Desserts into a single category, and created a super category labeled “main courses” that includes Burgers, Entrees, Pizza, Salads, Sandwiches, and Soups. We also excluded the toppings and ingredients category since restaurant reporting of these items was not consistent over the study period.

**Supplemental Information References**

1. Bleich SN, Wolfson JA, Jarlenski MP. Calorie changes in large chain restaurants: declines in new menu items but room for improvement. *Am J Prev Med.* 2016;50(1):e1-e8.

2. Jarlenski MP, Wolfson JA, Bleich SN. Macronutrient composition of menu offerings in fast food restaurants in the US. *Am J Prev Med.* 2016;51(4):e91-e97.

3. Bleich SN, Wolfson JA, Jarlenski MP, Block JP. Restaurants with calories displayed on menus had lower calorie counts compared to restaurants without such labels. *Health Aff.* 2015;34(11):1877-1884.

4. Cleveland LP, Simon D, Block JP. Compliance in 2017 With Federal Calorie Labeling in 90 Chain Restaurants and 10 Retail Food Outlets Prior to Required Implementation. *Am J Public Health.* 2018;108(8):1099-1102.

5. Patient protection and affordable care act, Public law(2010).

6. Moran AJ, Block JP, Goshev SG, Bleich SN, Roberto CA. Trends in nutrient content of children’s menu items in US chain restaurants. *Am J Prev Med.* 2017;52(3):284-291.

7. Bleich SN, Wolfson JA, Jarlenski MP. Calorie changes in chain restaurant menu items: implications for obesity and evaluations of menu labeling. *Am J Prev Med.* 2015;48(1):70-75.

8. MenuStat Methods. 2018; <http://menustat.org/Content/assets/pdfFile/MenuStat%20Data%20Completeness%20Documentation.pdf>. Accessed September 4, 2018.
