# Supplementary material for: Calorie and nutrient trends in large U.S. chain restaurants, 2012-2018
Source: PLoS One. 2020 Feb 10;15(2):e0228891. doi: 10.1371/journal.pone.0228891 (PMC7010289; doi:10.1371/journal.pone.0228891)
Supplement: S1 Table — (DOCX) [file pone.0228891.s002.docx]

**S1Table.**Characteristics of 66 Restaurants Included in Study

| **Restaurant** | **No. Items^a^** | **% Items^a^** | **No. Census divisions^b^** | **Implementation of calorie labeling^c^** |
| --- | --- | --- | --- | --- |
| ***Fast Food*** | **13,435** | **47.58** | **7.7** |  |
| Arby's | 170 | 0.60 | 9 | 2017 |
| Baskin Robbins | 549 | 1.94 | 9 | 2017 |
| Bojangles | 110 | 0.39 | 3 | 2017 |
| Burger King | 441 | 1.56 | 9 | 2018 |
| Carl's Jr. | 230 | 0.81 | 8 | 2017 |
| Checker's Drive-In/Rallys | 139 | 0.49 | 8 | 2018 |
| Chick-Fil-A | 128 | 0.45 | 9 | 2013 |
| Church's Chicken | 143 | 0.51 | 8 | 2018 |
| Culver's | 393 | 1.39 | 6 | 2017 |
| Dairy Queen | 650 | 2.30 | 9 | 2018 |
| Del Taco | 196 | 0.69 | 6 | 2017 |
| Dominos | 184 | 0.65 | 9 | - |
| Dunkin' Donuts | 1,745 | 6.18 | 9 | 2017 |
| Einstein Bros | 364 | 1.29 | 9 | 2018 |
| El Pollo Loco | 270 | 0.96 | 3 | 2017 |
| Five Guys | 16 | 0.06 | 9 | 2018 |
| Hardee's | 246 | 0.87 | 7 | 2018 |
| In-N-Out Burger | 49 | 0.17 | 3 | 2017 |
| Jack in the Box | 305 | 1.08 | 7 | 2017 |
| Jamba Juice | 439 | 1.55 | 9 | 2010 |
| KFC | 212 | 0.75 | 9 | 2017 |
| Krystal | 90 | 0.32 | 3 | 2017 |
| Little Caesars | 31 | 0.11 | 9 | 2017 |
| Long John Silver's | 88 | 0.31 | 9 | 2017 |
| McDonald's | 710 | 2.51 | 9 | 2012 |
| Panda Express | 148 | 0.52 | 9 | 2017 |
| Papa John's | 795 | 2.82 | 9 | 2018 |
| Popeyes | 83 | 0.29 | 9 | 2018 |
| Quiznos | 667 | 2.36 | 9 | 2017 |
| Sonic | 1,199 | 4.25 | 9 | 2017 |
| Steak 'N Shake | 562 | 1.99 | 8 | 2017 |
| Subway | 253 | 0.90 | 9 | 2017 |
| Taco Bell | 389 | 1.38 | 9 | 2017 |
| Tim Hortons | 528 | 1.87 | 8 | 2017 |
| Wendy's | 200 | 0.71 | 9 | 2017 |
| Whataburger | 252 | 0.89 | 4 | 2018 |
| White Castle | 461 | 1.63 | 5 | 2017 |
| ***Fast Casual*** | **6384** | **22.61** | **8.1** |  |
| Boston Market | 142 | 0.50 | 8 | 2017 |
| Captain D's | 111 | 0.39 | 8 | 2017 |
| Chipotle | 19 | 0.07 | 9 | 2017 |
| Ci Ci's Pizza | 54 | 0.19 | 8 | 2017 |
| Jason's Deli | 354 | 1.25 | 7 | 2017 |
| Jimmy John's | 40 | 0.14 | 9 | 22017 |
| Panera Bread | 384 | 1.36 | 9 | 2010 |
| Papa Murphy's | 236 | 0.84 | 7 | - |
| Qdoba | 31 | 0.11 | 9 | 2017 |
| Starbucks | 4,757 | 16.85 | 9 | 2013 |
| Zaxby's | 256 | 0.91 | 6 | 2018 |
| ***Full Service*** | **8,419** | **29.81** | **8.3** |  |
| Applebee's | 427 | 1.51 | 9 | 2017 |
| Bob Evans | 578 | 2.05 | 6 | 2017 |
| California Pizza Kitchen | 450 | 1.59 | 9 | 2017 |
| Chili's | 444 | 1.57 | 9 | 2017 |
| Denny's | 611 | 2.16 | 9 | 2017 |
| Friendly's | 539 | 1.91 | 3 | 2017 |
| Golden Corral | 673 | 2.38 | 9 | 2018 |
| IHOP | 730 | 2.59 | 9 | 2018 |
| LongHorn Steakhouse | 375 | 1.33 | 8 | 2018 |
| O'Charley's | 259 | 0.92 | 6 | 2018 |
| Olive Garden | 399 | 1.41 | 9 | 2018 |
| Outback Steakhouse | 283 | 1.00 | 9 | 2018 |
| PF Chang's | 328 | 1.16 | 9 | 2018 |
| Pizza Hut | 829 | 2.94 | 9 | 2018 |
| Red Lobster | 497 | 1.76 | 9 | 2018 |
| Romano's Macaroni Grill | 168 | 0.59 | 9 | 2018 |
| Ruby Tuesday | 277 | 0.98 | 9 | 2018 |
| TGI Friday's | 552 | 1.95 | 9 | 2018 |
| **TOTAL** | **28,238** | **100.0** | **7.9** |  |

^a^ Indicates the total number and percentage of items in the MenuStat data in 2012-2018.

^b^ Indicates the total number of 9 U.S. Census Divisions in which a restaurant has locations. Means are shown for numbers in bold.

**^c^** Indicates latest year reported for full implementation of menu calorie labeling. – represents non-compliance as of 2018.
