# Supplementary material for: Calorie and nutrient trends in large U.S. chain restaurants, 2012-2018
Source: PLoS One. 2020 Feb 10;15(2):e0228891. doi: 10.1371/journal.pone.0228891 (PMC7010289; doi:10.1371/journal.pone.0228891)
Supplement: S2 Table — (DOCX) [file pone.0228891.s003.docx]

| **Table S2.** Restaurant- and item-level characteristics of items on menus from 2012-2018, overall and by category | | | | | | | | | | | | | |
| --- | --- | --- | --- | --- | --- | --- | --- | --- | --- | --- | --- | --- | --- |
| **Menu Category** | ***n***  **(%)** | **National Chain** | **Restaurant Type** | | | **Year of menu calorie labeling implementation** | | | | **Children’s menu** | **Regional only item** | **Limited time offer** | **Shareable item** |
|  |  |  | **Fast Food**  (n=37) | **Fast Casual** (n=11) | **Full Service** (n=18) | **Pre-2015** | **2017** | **2018** | **No labeling** |  |  |  |  |
| **Overall**^a^ | 28,238 | 21,360 | 13,435 | 6,384 | 8,419 | 6,418 | 14,968 | 6,432 | 420 | 1,565 | 1,159 | 1,496 | 634 |
| Food^b^ | 54.4% | 48.4% | 51.4% | 24.6% | 81.7% | 12.9% | 62.0% | 75.2% | 99.5% | 62.2% | 77.0% | 46.8% | 70.0% |
| Beverages | 45.6% | 51.6% | 48.6% | 75.4% | 18.3% | 87.1% | 38.0% | 24.8% | 0.5% | 37.8% | 23.0% | 53.2% | 30.0% |
| **Food Category** | 15,357 | 10,344 | 6,912 | 1,569 | 6,876 | 831 | 9,274 | 4,834 | 418 | 974 | 892 | 700 | 444 |
| Appetizers  & sides | 11.2% | 11.0% | 7.7% | 15.3% | 13.8% | 3.9% | 11.1% | 13.4% | 3.3% | 19.8% | 9.5% | 4.3% | 49.8% |
| Main courses | 70.5% | 70.9% | 67.1% | 64.7% | 75.3% | 65.5% | 70.6% | 69.7% | 88.3% | 63.1% | 71.7% | 68.9% | 28.6% |
| Fried Potatoes | 2.4% | 1.7% | 3.7% | 0.4% | 1.5% | 1.2% | 2.7% | 2.0% | 0.0% | 5.0% | 2.7% | 2.1% | 6.1% |
| Desserts &  baked goods | 15.9% | 16.4% | 21.5% | 19.6% | 9.4% | 29.5% | 15.6% | 14.9% | 8.4% | 12.0% | 16.0% | 24.7% | 15.5% |
| **Main course subcategory** | 10,829 | 7,329 | 4,637 | 1,015 | 5,177 | 544 | 6,547 | 3,369 | 369 | 615 | 640 | 482 | 127 |
| Burgers | 8.4% | 4.6% | 13.8% | 0.3% | 5.2% | 5.3% | 8.8% | 9.1% | 0.0% | 12.0% | 8.8% | 5.6% | 4.7% |
| Entrees | 37.5% | 38.6% | 16.2% | 24.3% | 59.2% | 30.1% | 34.0% | 49.1% | 5.4% | 62.1% | 20.8% | 35.9% | 47.2% |
| Pizza | 14.7% | 18.3% | 13.4% | 24.4% | 13.9% | 0.6% | 11.8% | 14.5% | 89.2% | 5.7% | 25.6% | 13.1% | 37.8% |
| Salads | 8.4% | 7.9% | 6.5% | 13.1% | 9.2% | 15.6% | 8.9% | 6.7% | 3.5% | 3.3% | 2.2% | 9.1% | 4.7% |
| Sandwiches | 26.8% | 27.1% | 46.4% | 28.7% | 8.9% | 41.4% | 31.4% | 18.2% | 1.9% | 13.3% | 36.1% | 31.1% | 4.7% |
| Soups | 4.2% | 3.7% | 3.7% | 9.2% | 3.7% | 7.0% | 5.1% | 2.5% | 0.0% | 3.6% | 6.6% | 5.2% | 0.8% |

*Note.* Major row values are *n* of all menu items. Minor row values are proportions (%) of the major row column totals, unless otherwise indicated.

^a^ Included all menu categories except toppings & ingredients.

^b^ Included all menu categories except beverages and toppings & ingredients.
