# Supplementary material for: Calorie and nutrient trends in large U.S. chain restaurants, 2012-2018
Source: PLoS One. 2020 Feb 10;15(2):e0228891. doi: 10.1371/journal.pone.0228891 (PMC7010289; doi:10.1371/journal.pone.0228891)
Supplement: S3 Table — (DOCX) [file pone.0228891.s004.docx]

**S3 Table.** Items missing nutrients on menus in 66 U.S. restaurants from 2012-2018, overall and by category

| **Menu Category** | **Missing calories** | **Missing saturated fat** | **Missing**  **trans fat** | **Missing unsaturated fat** | **Missing**  **sugar** | **Missing non-sugar carbohydrates** | **Missing**  **protein** | **Missing**  **sodium** |
| --- | --- | --- | --- | --- | --- | --- | --- | --- |
| Overall^a^ | 8,025 | 8,721 | 8,721 | 10,665 | 10,428 | 8,022 | 8,221 | 7,971 |
| Food^b^ | 29.60% | 29.93% | 29.93% | 39.85% | 39.88% | 29.82% | 29.70% | 29.93% |
| Beverage | 70.40% | 70.07% | 70.07% | 60.15% | 60.12% | 70.18% | 70.30% | 70.07% |
| **Food Category** | 2,375 | 2,610 | 2,610 | 4,250 | 4,159 | 2,392 | 2,442 | 2,386 |
| Appetizers & Sides | 8.55% | 9.43% | 9.43% | 11.20% | 12.00% | 9.82% | 9.38% | 9.35% |
| Main Courses | 70.95% | 72.95% | 72.95% | 72.99% | 74.15% | 71.57% | 72.03% | 71.88% |
| Fried Potatoes | 0.55% | 0.69% | 0.69% | 1.32% | 0.99% | 0.63% | 0.61% | 0.59% |
| Desserts & Baked Goods | 19.96% | 16.93% | 16.93% | 14.49% | 12.86% | 17.98% | 17.98% | 18.19% |
| **Main course subcategory** | 1,685 | 1,904 | 1,904 | 3,102 | 3,084 | 1,712 | 1,759 | 1,715 |
| Burgers | 8.72% | 8.72% | 8.72% | 8.96% | 6.74% | 8.18% | 7.79% | 8.05% |
| Entrees | 52.28% | 53.41% | 53.41% | 52.87% | 56.71% | 51.64% | 52.02% | 52.30% |
| Pizza | 4.93% | 5.30% | 5.30% | 4.16% | 5.54% | 6.02% | 5.69% | 5.42% |
| Salads | 4.69% | 5.09% | 5.09% | 7.80% | 8.56% | 5.43% | 5.80% | 5.36% |
| Sandwiches | 27.42% | 25.58% | 25.58% | 23.40% | 19.20% | 26.87% | 26.89% | 27.06% |
| Soups | 1.96% | 1.89% | 1.89% | 2.80% | 3.24% | 1.87% | 1.82% | 1.81% |

*Note.* Major row values are *n* of all menu items. Minor row values are proportions (%) of the major row column totals, unless otherwise indicated.

^a^ Included all menu categories except toppings & ingredients.

^b^ Included all menu categories except beverages and toppings & ingredients.
