# Supplementary material for: Calorie and nutrient trends in large U.S. chain restaurants, 2012-2018
Source: PLoS One. 2020 Feb 10;15(2):e0228891. doi: 10.1371/journal.pone.0228891 (PMC7010289; doi:10.1371/journal.pone.0228891)
Supplement: S4 Table — (DOCX) [file pone.0228891.s005.docx]

**S4 Table.** Predicted mean per-item calories, saturated fat, trans fat, unsaturated fat, sugar, non-sugar carbohydrates, protein and sodium for common items and for newly introduced items

| **Menu Category** | **n** | **Means** | | | | | | | **p-value for trend** | **2012-2018** | |
| --- | --- | --- | --- | --- | --- | --- | --- | --- | --- | --- | --- |
|  |  | **2012** | **2013** | **2014** | **2015** | **2016** | **2017** | **2018** |  | **Change** | **p-value** |
| **Common items: Overall^a^** | | | | | | | | | |  |  |
| Calories (kcal) | 4284 | 400 | 406 | 407 | 407 | 406 | 413 | 412 | 0.16 | 11 kcal | 0.20 |
| Saturated fat (g) | 4184 | 7.0 | 7.0 | 7.0 | 7.0 | 7.0 | 7.1 | 7.2 | 0.34 | 0.2 g | 0.35 |
| Trans fat (g) | 3533 | 0.3 | 0.3 | 0.3 | 0.3 | 0.3 | 0.3 | 0.2 | 0.25 | 0.0 g | 0.26 |
| Unsaturated fat (g) | 3523 | 10.1 | 10.0 | 10.0 | 10.0 | 9.9 | 10.0 | 10.0 | 0.85 | -0.1 g | 0.57 |
| Sugar (g) | 3529 | 24.1 | 24.0 | 24.0 | 23.8 | 23.8 | 24.0 | 24.9 | 0.54 | 0.8 g | 0.34 |
| Non-sugar carbohydrates (g) | 3509 | 21.6 | 21.6 | 21.9 | 21.9 | 22.5 | 22.5 | 21.9 | 0.17 | 0.3 g | 0.49 |
| Protein (g) | 4164 | 14.5 | 14.8 | 14.7 | 14.7 | 14.5 | 14.8 | 14.6 | 0.93 | 0.1 g | 0.71 |
| Sodium (mg) | 4206 | 664 | 683 | 690 | 687 | 687 | 705 | 710 | 0.14 | 46 mg | 0.16 |
|  | **n** |  | **New in 2013** | **New in 2014** | **New in 2015** | **New in 2016** | **New in 2017** | **New in 2018** | **p-value for trend** | **2012-2018** | |
|  |  |  |  |  |  |  |  |  |  | **Change** | **p-value** |
| **Newly introduced items: Overall^a^** |  |  |  |  |  |  |  |  |  |  |  |
| Calories (kcal) | **23954** |  | **477** | **486** | **455** | **458** | **428** | **357** | **0.02** | **-120 kcal** | **0.01** |
| Saturated fat (g) | 22961 |  | 8.2 | 8.5 | 8.0 | 7.5 | 7.6 | 4.8 | 0.06 | **-3.4 g** | **0.00** |
| Trans fat (g) | 21689 |  | 0.2 | 0.3 | 0.2 | 0.2 | 0.2 | 0.1 | 0.09 | -0.1 g | 0.15 |
| Unsaturated fat (g) | **21667** |  | **12.2** | **11.7** | **11.5** | **12.4** | **10.1** | **7.8** | **0.04** | **-4.5 g** | **0.02** |
| Sugar (g) | 21971 |  | 32.7 | 33.5 | 31.8 | 29.5 | 32.8 | 31.2 | 0.71 | -1.5 g | 0.69 |
| Non-sugar carbohydrates (g) | 21921 |  | 25.6 | 24.3 | 22.1 | 21.2 | 33.5 | 15.3 | 0.69 | **-10.3 g** | **0.02** |
| Protein (g) | **23471** |  | **17.0** | **16.8** | **15.9** | **16.9** | **14.3** | **12.7** | **0.02** | **-4.3 g** | **0.04** |
| Sodium (mg) | 23686 |  | 735 | 752 | 717 | 778 | 669 | 585 | 0.26 | -150 mg | 0.24 |

*Note.* All estimates are adjusted for restaurant type, whether the restaurant is a national chain, the year the restaurant began labeling their menus with calories, and whether the item is categorized as a kid’s item, shareable, regional or offered for a limited time. Estimates for common items included item fixed effects.

^a^ Included all menu categories except toppings & ingredients.
