# Supplementary material for: Calorie and nutrient trends in large U.S. chain restaurants, 2012-2018
Source: PLoS One. 2020 Feb 10;15(2):e0228891. doi: 10.1371/journal.pone.0228891 (PMC7010289; doi:10.1371/journal.pone.0228891)
Supplement: S5 Table — (DOCX) [file pone.0228891.s006.docx]

**S5 Table.** Predicted mean per-item calories, saturated fat, trans fat, unsaturated fat, sugar, non-sugar carbohydrates, protein and sodium by category for common items, 2012-2018.

| **Menu Category** | ***n*** | **Means** | | | | | | | ***p*-value for trend** | **2012-2018** | |
| --- | --- | --- | --- | --- | --- | --- | --- | --- | --- | --- | --- |
|  |  | **2012** | **2013** | **2014** | **2015** | **2016** | **2017** | **2018** |  | ***Change*** | ***p-value*** |
| **Appetizers & Sides** |  |  |  |  |  |  |  |  |  |  |  |
| Calories (kcal) | 359 | 319 | 321 | 325 | 325 | 330 | 339 | 336 | 0.13 | 17 kcal | 0.17 |
| Saturated fat (g) | 342 | 4.7 | 4.6 | 4.6 | 4.7 | 4.8 | 4.8 | 4.6 | 0.87 | -0.1 g | 0.70 |
| Trans fat (g) | 258 | 0.2 | 0.2 | 0.2 | 0.2 | 0.2 | 0.2 | 0.1 | 0.30 | -0.1 g | 0.21 |
| Unsaturated fat (g) | 257 | 11.8 | 11.8 | 12.2 | 12.1 | 12.1 | 12.2 | 12.0 | 0.67 | 0.2 g | 0.81 |
| Sugar (g) | **255** | **5.7** | **5.8** | **5.6** | **5.5** | **5.4** | **5.3** | **5.0** | **0.03** | **-0.7 g** | **0.04** |
| Non-sugar carbohydrates (g) | 252 | 25.3 | 25.2 | 25.4 | 25.9 | 33.2 | 26.1 | 26.3 | 0.10 | 1.0 g | 0.10 |
| Protein (g) | 349 | 10.7 | 11.0 | 11.0 | 11.0 | 10.6 | 11.0 | 10.9 | 0.89 | 0.3 g | 0.69 |
| Sodium (mg) | **348** | **664** | **690** | **710** | **708** | **720** | **757** | **777** | **0.01** | **112 mg** | **0.01** |
| **Main courses^a^** |  |  |  |  |  |  |  |  |  |  |  |
| Calories (kcal) | 1869 | 505 | 516 | 520 | 522 | 516 | 527 | 515 | 0.40 | 10 kcal | 0.52 |
| Saturated fat (g) | 1808 | 9.2 | 9.2 | 9.2 | 9.3 | 9.2 | 9.4 | 9.4 | 0.46 | 0.1 g | 0.58 |
| Trans fat (g) | 1450 | 0.4 | 0.4 | 0.4 | 0.4 | 0.4 | 0.4 | 0.3 | 0.25 | -0.1 g | 0.12 |
| Unsaturated fat (g) | 1443 | 16.0 | 15.8 | 15.7 | 15.9 | 15.6 | 15.8 | 15.6 | 0.53 | -0.4 g | 0.28 |
| Sugar (g) | 1424 | 6.4 | 6.3 | 6.5 | 6.6 | 6.5 | 6.6 | 6.6 | 0.19 | 0.2 g | 0.28 |
| Non-sugar carbohydrates (g) | 1423 | 32.9 | 32.8 | 33.4 | 33.4 | 33.3 | 34.3 | 32.6 | 0.66 | -0.3 g | 0.73 |
| Protein (g) | 1835 | 25.3 | 26.0 | 25.8 | 25.6 | 25.3 | 25.7 | 25.3 | 0.79 | 0.1 g | 0.93 |
| Sodium (mg) | 1810 | 1173 | 1211 | 1221 | 1216 | 1212 | 1245 | 1244 | 0.28 | 71 mg | 0.29 |
| **Burgers** |  |  |  |  |  |  |  |  |  |  |  |
| Calories (kcal) | 221 | 612 | 610 | 628 | 628 | 616 | 600 | 599 | 0.41 | -13 kcal | 0.54 |
| Saturated fat (g) | 214 | 13.7 | 13.9 | 13.8 | 13.7 | 13.5 | 13.4 | 12.9 | 0.19 | -0.8 g | 0.32 |
| Trans fat (g) | 192 | 1.0 | 1.1 | 1.1 | 1.1 | 1.3 | 1.2 | 1.1 | 0.29 | 0.0 g | 0.71 |
| Unsaturated fat (g) | 191 | 19.6 | 19.4 | 19.2 | 20.0 | 19.9 | 19.8 | 19.2 | 0.97 | -0.4 g | 0.76 |
| Sugar (g) | 199 | 7.0 | 7.1 | 7.5 | 7.4 | 7.6 | 7.5 | 7.7 | 0.06 | 0.7 g | 0.08 |
| Non-sugar carbohydrates (g) | 199 | 31.1 | 31.5 | 31.6 | 31.6 | 31.0 | 30.8 | 30.8 | 0.54 | -0.3 g | 0.79 |
| Protein (g) | 220 | 31.1 | 30.9 | 30.5 | 30.3 | 30.7 | 30.7 | 30.6 | 0.51 | -0.5 g | 0.51 |
| Sodium (mg) | 218 | 1062 | 1089 | 1131 | 1137 | 1140 | 1082 | 1175 | 0.26 | 113 mg | 0.15 |
| **Entrees** |  |  |  |  |  |  |  |  |  |  |  |
| Calories (kcal) | 633 | 573 | 608 | 615 | 618 | 606 | 620 | 610 | 0.39 | 37 kcal | 0.38 |
| Saturated fat (g) | 592 | 9.9 | 9.9 | 9.9 | 10.1 | 10.1 | 10.3 | 10.5 | 0.22 | 0.6 g | 0.24 |
| Trans fat (g) | 418 | 0.5 | 0.6 | 0.5 | 0.4 | 0.4 | 0.4 | 0.4 | 0.05 | -0.2 g | 0.05 |
| Unsaturated fat (g) | 417 | 19.5 | 19.1 | 19.7 | 19.8 | 18.8 | 18.8 | 19.0 | 0.23 | -0.5 g | 0.28 |
| Sugar (g) | 382 | 8.0 | 7.8 | 8.1 | 8.2 | 8.0 | 8.0 | 8.5 | 0.25 | 0.5 g | 0.28 |
| Non-sugar carbohydrates (g) | 381 | 33.6 | 33.5 | 35.0 | 35.0 | 35.4 | 34.0 | 33.4 | 0.94 | -0.3 g | 0.88 |
| Protein (g) | 608 | 30.9 | 32.7 | 32.7 | 32.2 | 31.5 | 31.3 | 31.2 | 0.66 | 0.3 g | 0.91 |
| Sodium (mg) | 593 | 1278 | 1381 | 1403 | 1389 | 1372 | 1443 | 1446 | 0.32 | 168 mg | 0.30 |
| **Pizza** |  |  |  |  |  |  |  |  |  |  |  |
| Calories (kcal) | 211 | 311 | 311 | 308 | 316 | 306 | 397 | 310 | 0.45 | -1 kcal | 0.96 |
| Saturated fat (g) | 206 | 6.0 | 6.0 | 6.0 | 6.2 | 6.0 | 7.7 | 6.2 | 0.34 | 0.2 g | 0.58 |
| Trans fat (g) | 203 | 0.0 | 0.0 | 0.0 | 0.0 | 0.0 | 0.1 | 0.0 | 0.51 | 0.0 g | 1.00 |
| Unsaturated fat (g) | 198 | 7.6 | 7.5 | 7.6 | 7.8 | 7.5 | 9.4 | 7.4 | 0.51 | -0.1 g | 0.84 |
| Sugar (g) | 191 | 4.7 | 4.8 | 4.5 | 4.7 | 4.4 | 5.4 | 4.2 | 0.88 | -0.6 g | 0.35 |
| Non-sugar carbohydrates (g) | 191 | 26.5 | 26.4 | 26.6 | 27.0 | 26.3 | 37.4 | 27.4 | 0.33 | 0.9 g | 0.67 |
| Protein (g) | 211 | 14.1 | 14.0 | 13.8 | 14.2 | 13.7 | 17.9 | 13.6 | 0.56 | -0.5 g | 0.59 |
| Sodium (mg) | 211 | 754 | 745 | 737 | 752 | 722 | 888 | 726 | 0.73 | -27 mg | 0.69 |
| **Salads** |  |  |  |  |  |  |  |  |  |  |  |
| Calories (kcal) | 151 | 458 | 457 | 459 | 463 | 458 | 463 | 464 | 0.69 | 6 kcal | 0.65 |
| Saturated fat (g) | 146 | 7.7 | 7.6 | 7.2 | 7.2 | 7.2 | 7.2 | 7.4 | 0.43 | -0.2 g | 0.52 |
| Trans fat (g) | 102 | 0.2 | 0.2 | 0.2 | 0.2 | 0.2 | 0.1 | 0.1 | 0.19 | -0.1 g | 0.30 |
| Unsaturated fat (g) | 102 | 17.5 | 17.1 | 16.9 | 17.2 | 16.9 | 17.3 | 17.8 | 0.82 | 0.3 g | 0.76 |
| Sugar (g) | 98 | 7.1 | 7.1 | 7.2 | 8.0 | 8.0 | 8.4 | 8.4 | 0.19 | 1.3 g | 0.22 |
| Non-sugar carbohydrates (g) | 98 | 19.9 | 19.4 | 20.1 | 19.6 | 18.9 | 19.2 | 18.6 | 0.31 | -1.3 g | 0.23 |
| Protein (g) | 146 | 24.7 | 25.0 | 24.3 | 24.3 | 24.2 | 24.2 | 24.8 | 0.52 | 0.0 g | 0.96 |
| Sodium (mg) | 142 | 961 | 960 | 949 | 925 | 941 | 944 | 949 | 0.65 | -12 mg | 0.73 |
| **Sandwiches** |  |  |  |  |  |  |  |  |  |  |  |
| Calories (kcal) | 502 | 531 | 527 | 525 | 524 | 528 | 519 | 522 | 0.44 | -10 kcal | 0.38 |
| Saturated fat (g) | 501 | 9.0 | 8.9 | 8.9 | 8.9 | 8.9 | 8.8 | 9.0 | 0.86 | 0.1 g | 0.82 |
| Trans fat (g) | 419 | 0.2 | 0.3 | 0.3 | 0.3 | 0.3 | 0.3 | 0.2 | 0.20 | -0.1 g | 0.12 |
| Unsaturated fat (g) | 419 | 16.7 | 16.5 | 15.8 | 15.8 | 16.1 | 15.8 | 16.1 | 0.51 | -0.6 g | 0.47 |
| Sugar (g) | 454 | 5.7 | 5.6 | 5.7 | 5.7 | 5.7 | 5.6 | 5.5 | 0.38 | -0.2 g | 0.22 |
| Non-sugar carbohydrates (g) | 454 | 40.5 | 40.3 | 40.7 | 40.5 | 40.6 | 40.0 | 39.7 | 0.52 | -0.7 g | 0.51 |
| Protein (g) | 500 | 24.3 | 24.7 | 24.5 | 24.4 | 24.3 | 24.1 | 24.3 | 0.52 | 0.0 g | 0.95 |
| Sodium (mg) | 501 | 1333 | 1312 | 1303 | 1296 | 1304 | 1297 | 1296 | 0.32 | -37 mg | 0.19 |
| **Soup** |  |  |  |  |  |  |  |  |  |  |  |
| Calories (kcal) | 151 | 292 | 301 | 304 | 304 | 304 | 306 | 310 | 0.22 | 18 kcal | 0.17 |
| Saturated fat (g) | 149 | 7.1 | 7.1 | 7.2 | 7.4 | 7.2 | 7.2 | 7.1 | 0.99 | 0.0 g | 0.98 |
| Trans fat (g) | 116 | 0.3 | 0.3 | 0.2 | 0.2 | 0.2 | 0.3 | 0.3 | 0.71 | 0.1 g | 0.55 |
| Unsaturated fat (g) | 116 | 7.9 | 8.0 | 8.2 | 8.2 | 8.1 | 7.9 | 7.8 | 0.69 | -0.1 g | 0.86 |
| Sugar (g) | 100 | 5.0 | 4.9 | 5.1 | 5.3 | 5.4 | 5.4 | 5.4 | 0.09 | 0.4 g | 0.24 |
| Non-sugar carbohydrates (g) | 100 | 23.9 | 23.9 | 24.4 | 24.1 | 24.4 | 24.8 | 24.6 | 0.26 | 0.7 g | 0.30 |
| Protein (g) | 150 | 13.1 | 13.5 | 13.5 | 13.2 | 13.1 | 13.7 | 14.1 | 0.35 | 1.0 g | 0.20 |
| Sodium (mg) | 145 | 1172 | 1268 | 1299 | 1315 | 1320 | 1322 | 1383 | 0.06 | 211 mg | 0.08 |
| **Fried Potatoes** |  |  |  |  |  |  |  |  |  |  |  |
| Calories (kcal) | 163 | 429 | 441 | 443 | 421 | 451 | 451 | 444 | 0.43 | 15 kcal | 0.40 |
| Saturated fat (g) | 161 | 5.7 | 5.9 | 5.8 | 5.4 | 5.9 | 5.8 | 5.8 | 0.93 | 0.1 g | 0.85 |
| Trans fat (g) | 134 | 0.4 | 0.4 | 0.4 | 0.3 | 0.4 | 0.3 | 0.4 | 0.54 | 0.0 g | 0.70 |
| Unsaturated fat (g) | 134 | 18.8 | 19.1 | 19.3 | 18.3 | 19.7 | 19.9 | 19.7 | 0.28 | 0.9 g | 0.29 |
| Sugar (g) | 142 | 0.7 | 0.8 | 0.8 | 0.7 | 0.8 | 0.8 | 0.7 | 0.77 | 0.0 g | 0.68 |
| Non-sugar carbohydrates (g) | 142 | 46.4 | 47.1 | 47.7 | 45.3 | 47.8 | 47.5 | 46.2 | 0.98 | -0.2 g | 0.90 |
| Protein (g) | 162 | 5.8 | 6.0 | 6.1 | 5.8 | 6.5 | 6.5 | 6.3 | 0.33 | 0.5 g | 0.34 |
| Sodium (mg) | **161** | **665** | **702** | **721** | **706** | **723** | **724** | **775** | **0.04** | **110 mg** | **0.04** |
| **Desserts and Baked Goods** |  |  |  |  |  |  |  |  |  |  |  |
| Calories (kcal) | 658 | 379 | 382 | 382 | 381 | 380 | 393 | 408 | 0.28 | 29 kcal | 0.26 |
| Saturated fat (g) | 652 | 8.4 | 8.5 | 8.5 | 8.4 | 8.6 | 8.8 | 9.5 | 0.13 | 1.0 g | 0.15 |
| Trans fat (g) | 591 | 0.3 | 0.3 | 0.3 | 0.3 | 0.2 | 0.2 | 0.2 | 0.26 | 0.0 g | 0.46 |
| Unsaturated fat (g) | 591 | 7.5 | 7.5 | 7.4 | 7.3 | 7.5 | 7.7 | 7.8 | 0.22 | 0.3 g | 0.31 |
| Sugar (g) | 612 | 31.7 | 31.6 | 31.5 | 30.8 | 30.2 | 31.6 | 33.5 | 0.79 | 1.8 g | 0.62 |
| Non-sugar carbohydrates (g) | 611 | 18.7 | 18.5 | 18.7 | 18.9 | 18.8 | 19.6 | 19.8 | 0.05 | 1.1 g | 0.13 |
| Protein (g) | 646 | 6.8 | 6.9 | 6.9 | 6.9 | 7.0 | 7.3 | 7.6 | 0.12 | 0.7 g | 0.13 |
| Sodium (mg) | 655 | 273 | 276 | 277 | 278 | 275 | 284 | 287 | 0.13 | 14 mg | 0.16 |

*Note.* Boldface indicates statistical significance at *p*<0.05. The n indicates total number of items available in all years for that category. All estimates included item fixed effects and are adjusted for whether the item is categorized as shareable, regional or offered for a limited time.

^a^ Included burgers, entrees, pizza, salads, sandwiches and soup menu categories.
