# Supplementary material for: Calorie and nutrient trends in large U.S. chain restaurants, 2012-2018
Source: PLoS One. 2020 Feb 10;15(2):e0228891. doi: 10.1371/journal.pone.0228891 (PMC7010289; doi:10.1371/journal.pone.0228891)
Supplement: S6 Table — (DOCX) [file pone.0228891.s007.docx]

**S6 Table.** Predicted mean per-item calories, saturated fat, trans fat, unsaturated fat, sugar, non-sugar carbohydrates, protein and sodium by category for newly introduced items, 2013-2018

| **Menu Category** | ***n*** | **Means** | | | | | | ***p*-value for trend** | **2013-2018** | |
| --- | --- | --- | --- | --- | --- | --- | --- | --- | --- | --- |
|  |  | **New in 2013** | **New in 2014** | **New in 2015** | **New in 2016** | **New in 2017** | **New in 2018** |  | **Change** | **p-value** |
| **Appetizers & Sides** |  |  |  |  |  |  |  |  |  |  |
| Calories (kcal) | 1363 | 532 | 509 | 454 | 482 | 574 | 537 | 0.46 | 5 kcal | 0.95 |
| Saturated fat (g) | 1315 | 8.0 | 9.2 | 7.5 | 7.9 | 9.2 | 8.4 | 0.83 | 0.4 g | 0.79 |
| Trans fat (g) | 1163 | 0.3 | 0.3 | 0.3 | 0.1 | 0.3 | 0.3 | 1.00 | 0.0 g | 0.98 |
| Unsaturated fat (g) | 1162 | 22.2 | 19.7 | 19.3 | 21.1 | 25.0 | 22.9 | 0.27 | 0.7 g | 0.89 |
| Sugar (g) | **1154** | **7.1** | **6.1** | **5.5** | **8.1** | **10.3** | **9.3** | **0.03** | 2.3 g | 0.27 |
| Non-sugar carbohydrates (g) | 1148 | 36.9 | 37.8 | 29.8 | 21.9 | 38.9 | 30.5 | 0.69 | -6.4 g | 0.23 |
| Protein (g) | 1325 | 19.9 | 20.8 | 16.5 | 26.8 | 25.2 | 28.6 | 0.07 | 8.7 g | 0.21 |
| Sodium (mg) | 1331 | 1162 | 1239 | 1218 | 1248 | 1415 | 1513 | 0.11 | 351 mg | 0.18 |
| **Main courses^a^** |  |  |  |  |  |  |  |  |  |  |
| Calories (kcal) | 8960 | 640 | 632 | 622 | 640 | 533 | 561 | 0.10 | -80 kcal | 0.15 |
| Saturated fat (g) | 8662 | 10.6 | 11.0 | 10.7 | 10.9 | 10.9 | 9.6 | 0.57 | -1.0 g | 0.35 |
| Trans fat (g) | 7820 | 0.4 | 0.5 | 0.4 | 0.3 | 0.3 | 0.4 | 0.24 | -0.3 g | 0.75 |
| Unsaturated fat (g) | 7808 | 22.5 | 21.2 | 20.7 | 21.8 | 16.7 | 18.6 | 0.09 | -3.9 g | 0.11 |
| Sugar (g) | 7894 | 12.3 | 12.0 | 11.7 | 11.3 | 8.5 | 9.9 | 0.13 | -2.3 g | 0.29 |
| Non-sugar carbohydrates (g) | 7878 | 47.4 | 41.7 | 42.7 | 42.1 | 78.0 | 40.0 | 0.64 | -7.4 g | 0.16 |
| Protein (g) | 8777 | 31.2 | 31.0 | 30.2 | 29.6 | 25.9 | 28.3 | 0.12 | -2.9 g | 0.32 |
| Sodium (mg) | 8826 | 1524 | 1457 | 1407 | 1462 | 1215 | 1351 | 0.13 | -172 mg | 0.26 |
| **Burgers** |  |  |  |  |  |  |  |  |  |  |
| Calories (kcal) | **690** | **981** | **817** | **846** | **883** | **919** | **752** | **0.02** | **-229 kcal** | **0.01** |
| Saturated fat (g) | 663 | 18.1 | 14.6 | 17.0 | 20.2 | 35.3 | 16.8 | 0.39 | -1.3 g | 0.54 |
| Trans fat (g) | 571 | 1.3 | 1.2 | 1.5 | 1.5 | 1.6 | 1.2 | 0.62 | 0.0 g | 0.89 |
| Unsaturated fat (g) | 569 | 31.7 | 25.6 | 28.9 | 33.4 | 26.0 | 26.4 | 0.18 | -5.3 g | 0.09 |
| Sugar (g) | 635 | 24.4 | 15.6 | 10.6 | 15.3 | 9.1 | 16.4 | 0.19 | -8.0 g | 0.10 |
| Non-sugar carbohydrates (g) | 632 | 75.7 | 65.4 | 61.8 | 26.8 | 177.7 | 25.7 | 0.88 | -50.0 g | 0.07 |
| Protein (g) | 685 | 39.4 | 33.4 | 37.7 | 38.9 | 44.7 | 35.5 | 0.55 | -3.9 g | 0.29 |
| Sodium (mg) | 684 | 1698 | 1582 | 1677 | 1564 | 1954 | 1666 | 0.60 | -32 mg | 0.91 |
| **Entrees** |  |  |  |  |  |  |  |  |  |  |
| Calories (kcal) | 3429 | 714 | 727 | 744 | 698 | 643 | 656 | 0.48 | -58 kcal | 0.62 |
| Saturated fat (g) | 3253 | 11.3 | 11.9 | 12.8 | 11.3 | 10.9 | 11.1 | 0.70 | -0.2 g | 0.92 |
| Trans fat (g) | 2809 | 0.5 | 0.6 | 0.5 | 0.3 | 0.3 | 0.4 | 0.17 | -0.1 g | 0.48 |
| Unsaturated fat (g) | 2802 | 26.9 | 27.9 | 26.0 | 24.2 | 23.6 | 23.1 | 0.39 | -3.8 g | 0.41 |
| Sugar (g) | 2768 | 16.8 | 19.6 | 18.4 | 14.8 | 14.6 | 14.4 | 0.24 | -2.4 g | 0.54 |
| Non-sugar carbohydrates (g) | 2767 | 55.3 | 51.2 | 53.2 | 42.7 | 120.5 | 39.8 | 0.71 | -15.4 g | 0.11 |
| Protein (g) | 3337 | 39.6 | 34.8 | 38.6 | 31.6 | 34.1 | 35.7 | 0.39 | -3.8 g | 0.45 |
| Sodium (mg) | 3357 | 1692 | 1556 | 1644 | 1568 | 1421 | 1588 | 0.62 | -104 mg | 0.71 |
| **Pizza** |  |  |  |  |  |  |  |  |  |  |
| Calories (kcal) | 1380 | 370 | 435 | 388 | 351 | 305 | 301 | 0.19 | -69 kcal | 0.40 |
| Saturated fat (g) | 1360 | 7.3 | 9.8 | 7.0 | 6.0 | 5.7 | 5.7 | 0.19 | -1.6 g | 0.36 |
| Trans fat (g) | 1337 | 0.0 | 0.2 | 0.1 | 0.1 | 0.1 | 0.1 | 0.40 | **0.1 g** | **0.01** |
| Unsaturated fat (g) | 1336 | 9.5 | 13.2 | 10.6 | 7.8 | 7.7 | 7.0 | 0.09 | -2.5 g | 0.22 |
| Sugar (g) | 1301 | 5.1 | 4.6 | 5.2 | 5.0 | 3.2 | 3.6 | 0.09 | -1.5 g | 0.33 |
| Non-sugar carbohydrates (g) | 1291 | 33.8 | 39.2 | 36.5 | 35.9 | 29.7 | 30.2 | 0.34 | -3.6 g | 0.67 |
| Protein (g) | 1358 | 16.3 | 20.8 | 16.1 | 15.2 | 12.8 | 12.8 | 0.15 | -3.5 g | 0.39 |
| Sodium (mg) | 1368 | 890 | 978 | 910 | 803 | 689 | 643 | 0.09 | -247 mg | 0.23 |
| **Salads** |  |  |  |  |  |  |  |  |  |  |
| Calories (kcal) | 757 | 437 | 539 | 462 | 542 | 455 | 549 | 0.31 | **113 kcal** | **0.03** |
| Saturated fat (g) | 736 | 7.2 | 9.1 | 6.5 | 7.8 | 7.2 | 7.7 | 0.65 | 0.5 g | 0.56 |
| Trans fat (g) | 634 | 0.2 | 0.4 | 0.1 | 0.2 | 0.2 | 0.1 | 0.09 | -0.1 g | 0.41 |
| Unsaturated fat (g) | 633 | 20.7 | 22.3 | 20.1 | 23.4 | 20.0 | 22.8 | 0.78 | 2.1 g | 0.48 |
| Sugar (g) | 622 | 9.6 | 8.9 | 11.1 | 11.8 | 8.0 | 13.0 | 0.11 | 3.4 g | 0.08 |
| Non-sugar carbohydrates (g) | **621** | **17.4** | **20.5** | **19.6** | **26.4** | **25.4** | **26.3** | **<0.01** | **8.9 g** | **<0.01** |
| Protein (g) | 731 | 24.9 | 29.3 | 26.2 | 27.3 | 26.0 | 27.5 | 0.79 | 2.6 g | 0.26 |
| Sodium (mg) | 741 | 972 | 1171 | 964 | 1129 | 1084 | 1262 | 0.17 | 290 mg | 0.03 |
| **Sandwiches** |  |  |  |  |  |  |  |  |  |  |
| Calories (kcal) | 2402 | 669 | 588 | 609 | 639 | 619 | 598 | 0.23 | **-71 kcal** | **0.04** |
| Saturated fat (g) | 2353 | 10.8 | 10.8 | 9.6 | 10.6 | 9.0 | 10.3 | 0.13 | -0.5 g | 0.43 |
| Trans fat (g) | 2197 | 0.3 | 0.4 | 0.3 | 0.2 | 0.2 | 0.4 | 0.64 | 0.2 g | 0.17 |
| Unsaturated fat (g) | 2192 | 22.5 | 17.2 | 19.1 | 22.0 | 18.5 | 19.0 | 0.15 | **-3.5 g** | **0.02** |
| Sugar (g) | 2286 | 8.6 | 6.9 | 8.4 | 9.0 | 7.7 | 7.3 | 0.67 | -1.3 g | 0.36 |
| Non-sugar carbohydrates (g) | 2285 | 52.8 | 46.7 | 48.8 | 46.1 | 55.5 | 45.6 | 0.27 | **-7.3 g** | **0.03** |
| Protein (g) | 2366 | 29.9 | 30.0 | 28.3 | 31.5 | 28.6 | 28.6 | 0.55 | -1.3 g | 0.44 |
| Sodium (mg) | 2375 | 1635 | 1533 | 1504 | 1621 | 1557 | 1489 | 0.30 | -147 mg | 0.11 |
| **Soup** |  |  |  |  |  |  |  |  |  |  |
| Calories (kcal) | 302 | 272 | 240 | 287 | 372 | 313 | 309 | 0.16 | 37 kcal | 0.36 |
| Saturated fat (g) | 297 | 6.7 | 4.9 | 6.9 | 7.1 | 6.7 | 5.2 | 0.73 | -1.6 g | 0.40 |
| Trans fat (g) | 276 | 0.1 | 0.4 | 0.1 | 0.4 | 0.2 | 0.1 | 0.60 | 0.0 g | 0.85 |
| Unsaturated fat (g) | 276 | 7.1 | 6.6 | 8.5 | 7.9 | 9.2 | 8.8 | 0.05 | 1.7 g | 0.26 |
| Sugar (g) | 282 | 5.9 | 4.7 | 5.8 | 3.0 | 5.6 | 7.3 | 0.78 | 1.4 g | 0.54 |
| Non-sugar carbohydrates (g) | 282 | 21.2 | 16.1 | 20.9 | 42.0 | 21.0 | 24.9 | 0.46 | 3.7 g | 0.39 |
| Protein (g) | **300** | **9.7** | **11.4** | **13.4** | **12.7** | **14.5** | **12.5** | **0.01** | 2.7 g | 0.08 |
| Sodium (mg) | 301 | 1230 | 1093 | 1169 | 1236 | 1237 | 1230 | 0.83 | 0 mg | 1.00 |
| **Fried Potatoes** |  |  |  |  |  |  |  |  |  |  |
| Calories (kcal) | 198 | 504 | 501 | 574 | 676 | 553 | 543 | 0.52 | 39 kcal | 0.60 |
| Saturated fat (g) | 193 | 8.0 | 5.7 | 10.6 | 10.9 | 7.2 | 7.9 | 0.97 | -0.1 g | 0.97 |
| Trans fat (g) | 181 | 0.2 | 0.2 | 0.3 | 0.2 | 0.4 | 0.7 | 0.10 | 0.5 g | 0.07 |
| Unsaturated fat (g) | 181 | 22.2 | 20.3 | 25.4 | 30.4 | 24.0 | 20.5 | 0.85 | -1.8 g | 0.56 |
| Sugar (g) | 182 | 6.4 | 4.5 | 2.6 | 2.3 | 6.2 | 1.6 | 0.49 | **-4.8 g** | **0.01** |
| Non-sugar carbohydrates (g) | 182 | 42.4 | 49.9 | 50.0 | 59.6 | 49.1 | 50.3 | 0.35 | 7.9 g | 0.27 |
| Protein (g) | 194 | 9.0 | 7.8 | 12.1 | 15.4 | 9.0 | 11.4 | 0.58 | 2.3 g | 0.53 |
| Sodium (mg) | **195** | **867** | **955** | **906** | **1411** | **1127** | **1458** | **0.01** | 591 mg | 0.05 |
| **Desserts and Baked Goods** |  |  |  |  |  |  |  |  |  |  |
| Calories (kcal) | **1787** | **563** | **558** | **451** | **435** | **473** | **452** | **0.04** | -111 kcal | 0.08 |
| Saturated fat (g) | 1764 | 13.1 | 13.6 | 8.7 | 10.0 | 11.9 | 11.1 | 0.27 | -2.1 g | 0.35 |
| Trans fat (g) | 1658 | 0.3 | 0.4 | 0.4 | 0.3 | 0.3 | 0.3 | 0.32 | 0.0 g | 0.70 |
| Unsaturated fat (g) | 1658 | 10.9 | 12.3 | 9.3 | 10.2 | 11.6 | 10.4 | 0.68 | -0.5 g | 0.77 |
| Sugar (g) | **1717** | **51.9** | **48.2** | **31.0** | **33.4** | **42.2** | **32.1** | **0.02** | **-19.8 g** | **0.01** |
| Non-sugar carbohydrates (g) | 1716 | 24.3 | 25.3 | 31.7 | 24.4 | 22.9 | 25.7 | 0.82 | 1.4 g | 0.55 |
| Protein (g) | **1772** | **9.6** | **9.2** | **9.2** | **6.5** | **7.6** | **7.6** | **<0.01** | **-2.0 g** | **0.02** |
| Sodium (mg) | 1772 | 406 | 406 | 450 | 361 | 329 | 423 | 0.58 | 17 mg | 0.80 |

*Note.* Boldface indicates statistical significance at *p*<0.05. The n indicates total number of items introduced in all years for that category. All estimates are adjusted for restaurant type, whether the restaurant is a national chain, the year the restaurant began labeling their menus with calories, and whether the item is categorized as a kid’s item, shareable, regional or offered for a limited time.

^a^ Included burgers, entrees, pizza, salads, sandwiches and soup menu categories.
