# Supplementary material for: Calorie and nutrient trends in large U.S. chain restaurants, 2012-2018
Source: PLoS One. 2020 Feb 10;15(2):e0228891. doi: 10.1371/journal.pone.0228891 (PMC7010289; doi:10.1371/journal.pone.0228891)
Supplement: S7 Table — (DOCX) [file pone.0228891.s008.docx]

**S7 Table.** Predicted calorie-adjusted per-item mean saturated fat, trans fat, unsaturated fat, sugar, non-sugar carbohydrates, protein and sodium for newly introduced items, 2013-2018.

| **Menu Category** | ***n*** | **Means** | | | | | | ***p*-value for trend** | **2013-2018** | |
| --- | --- | --- | --- | --- | --- | --- | --- | --- | --- | --- |
|  |  | **New in 2013** | **New in 2014** | **New in 2015** | **New in 2016** | **New in 2017** | **New in 2018** |  | **Change** | **p-value** |
| **Overall^a^** |  |  |  |  |  |  |  |  |  |  |
| Saturated fat (g) | 22961 | 7.4 | 7.7 | 7.7 | 7.2 | 7.9 | 6.5 | 0.46 | -0.9 g | 0.11 |
| Trans fat (g) | 21693 | 0.2 | 0.2 | 0.2 | 0.2 | 0.2 | 0.2 | 0.90 | 0.0 g | 0.66 |
| Unsaturated fat (g) | 21667 | 10.8 | 10.3 | 10.8 | 11.9 | 10.8 | 10.5 | 0.89 | -0.3 g | 0.61 |
| Sugar (g) | 21971 | 31.0 | 31.7 | 31.1 | 28.8 | 33.4 | 34.7 | 0.22 | 3.7 g | 0.25 |
| Non-sugar carbohydrates (g) | 21921 | 23.3 | 21.8 | 21.1 | 20.2 | 34.4 | 20.3 | 0.51 | -3.0 g | 0.34 |
| Protein (g) | 23471 | 15.7 | 15.3 | 15.4 | 16.3 | 14.8 | 15.8 | 0.88 | 0 g | 0.96 |
| Sodium (mg) | 23686 | 669 | 675 | 692 | 745 | 694 | 741 | 0.26 | 72 mg | 0.28 |
| **Food^b^** |  |  |  |  |  |  |  |  |  |  |
| Saturated fat (g) | 11934 | 9.8 | 10.7 | 10.1 | 10.2 | 11.7 | 10.1 | 0.39 | 0.3 g | 0.62 |
| Trans fat (g) | 10826 | 0.3 | 0.4 | 0.4 | 0.3 | 0.4 | 0.4 | 0.91 | 0.1 g | 0.31 |
| Unsaturated fat (g) | 10809 | 18.4 | 18.4 | 18.7 | 19.7 | 19.0 | 19.3 | 0.13 | 0.8 g | 0.21 |
| Sugar (g) | 10947 | 17.5 | 16.4 | 14.5 | 14.4 | 14.8 | 13.4 | 0.08 | -4.1 g | 0.09 |
| Non-sugar carbohydrates (g) | 10924 | 39.1 | 36.9 | 39.1 | 36.3 | 68.4 | 39.6 | 0.35 | 0.5 g | 0.89 |
| Protein (g) | 12068 | 24.9 | 25.2 | 24.9 | 25.2 | 24.8 | 27.1 | 0.38 | 2 g | 0.23 |
| Sodium (mg) | 12124 | 1234 | 1218 | 1217 | 1234 | 1215 | 1331 | 0.28 | 98 mg | 0.18 |
| **Beverage** |  |  |  |  |  |  |  |  |  |  |
| Saturated fat (g) | 11027 | 4.5 | 4.7 | 5.0 | 4.0 | 3.8 | 3.1 | 0.18 | -1.4 g | 0.10 |
| Trans fat (g) | 10867 | 0.0 | 0.0 | 0.1 | 0.0 | 0.0 | 0.1 | 0.88 | 0.0 g | 0.70 |
| Unsaturated fat (g) | 10858 | 2.8 | 3.2 | 3.0 | 2.9 | 2.7 | 2.2 | 0.22 | **-0.6 g** | **0.02** |
| Sugar (g) | 11024 | 46.0 | 44.6 | 46.9 | 48.8 | 49.4 | 55.6 | 0.09 | **9.6 g** | **0.02** |
| Non-sugar carbohydrates (g) | 10997 | 5.1 | 7.1 | 4.9 | 5.3 | 5.1 | 3.9 | 0.12 | -1.2 g | 0.07 |
| Protein (g) | **11403** | **5.9** | **5.2** | **5.3** | **5.1** | **4.8** | **4.4** | **0.01** | **-2 g** | **<0.01** |
| Sodium (mg) | 11562 | 71 | 134 | 141 | 137 | 172 | 144 | 0.10 | **73 mg** | **0.03** |

*Note.* Boldface indicates statistical significance at *p*<0.05. The n indicates total number of items introduced in all years for that category. All estimates are adjusted for restaurant type, whether the restaurant is a national chain, the year the restaurant began labeling their menus with calories, and whether the item is categorized as a kid’s item, shareable, regional or offered for a limited time.

^a^ Included all menu categories except toppings & ingredients.

^b^ Included all menu categories except beverages and toppings & ingredients.
