# Supplementary material for: Calorie and nutrient trends in large U.S. chain restaurants, 2012-2018
Source: PLoS One. 2020 Feb 10;15(2):e0228891. doi: 10.1371/journal.pone.0228891 (PMC7010289; doi:10.1371/journal.pone.0228891)
Supplement: S8 Table — Excluding items whose calories are ±50 calories different than the sum of their nutrient calories (N = 1149) or calories cannot be calculated due to missing nutrients (N = 559). (DOCX) [file pone.0228891.s009.docx]

**S8 Table.** Predicted mean per-item calories, saturated fat, trans fat, unsaturated fat, sugar, non-sugar carbohydrates, protein and sodium for newly introduced items, 2013-2018. Excluding items whose calories are ±50 calories different than the sum of their nutrient calories (N=1149) or calories cannot be calculated due to missing nutrients (N=559)

| **Menu Category** | ***n*** | **Means** | | | | | | ***p*-value for trend** | **2013-2018** | |
| --- | --- | --- | --- | --- | --- | --- | --- | --- | --- | --- |
|  |  | **New in 2013** | **New in 2014** | **New in 2015** | **New in 2016** | **New in 2017** | **New in 2018** |  | **Change** | **p-value** |
| **Overall^a^** |  |  |  |  |  |  |  |  |  |  |
| Calories (kcal) | **22246** | **474** | **486** | **446** | **446** | **422** | **364** | **0.03** | **-110 kcal** | **0.02** |
| Saturated fat (g) | 21682 | 8.3 | 8.7 | 7.8 | 7.3 | 7.5 | 5.1 | 0.06 | -3.2 g | 0.01 |
| Trans fat (g) | 20594 | 0.2 | 0.3 | 0.2 | 0.2 | 0.2 | 0.2 | 0.08 | -0.1 g | 0.18 |
| Unsaturated fat (g) | **20584** | **12.2** | **12.0** | **11.1** | **11.7** | **9.7** | **8.3** | **0.04** | **-3.9 g** | **0.04** |
| Sugar (g) | 20734 | 33.1 | 34.4 | 32.1 | 29.5 | 33.3 | 30.6 | 0.56 | -2.5 g | 0.50 |
| Non-sugar carbohydrates (g) | 20734 | 23.3 | 24.2 | 21.6 | 21.7 | 19.8 | 16.8 | 0.05 | -6.5 g | 0.07 |
| Protein (g) | **22246** | **17.2** | **17.0** | **15.4** | **16.5** | **13.9** | **13.1** | **0.01** | -4.1 g | 0.05 |
| Sodium (mg) | 22203 | 734 | 756 | 696 | 746 | 661 | 613 | 0.31 | -121 mg | 0.34 |
| **Food^b^** |  |  |  |  |  |  |  |  |  |  |
| Calories (kcal) | 11586 | 602 | 593 | 570 | 574 | 509 | 534 | 0.10 | -69 kcal | 0.18 |
| Saturated fat (g) | 11393 | 10.6 | 11.0 | 10.1 | 10.2 | 10.2 | 9.4 | 0.33 | -1.2 g | 0.26 |
| Trans fat (g) | 10417 | 0.4 | 0.5 | 0.4 | 0.3 | 0.3 | 0.3 | 0.24 | 0.0 g | 0.76 |
| Unsaturated fat (g) | 10409 | 19.8 | 19.2 | 18.8 | 19.2 | 15.6 | 17.7 | 0.21 | -2.1 g | 0.33 |
| Sugar (g) | **10494** | **18.5** | **17.2** | **14.3** | **14.1** | **12.6** | **11.8** | **0.01** | **-6.8 g** | **0.02** |
| Non-sugar carbohydrates (g) | 10494 | 39.4 | 38.2 | 38.6 | 36.1 | 35.1 | 34.6 | 0.19 | -4.8 g | 0.19 |
| Protein (g) | 11586 | 26.0 | 25.6 | 24.4 | 25.2 | 22.0 | 24.8 | 0.34 | -1.3 g | 0.63 |
| Sodium (mg) | 11553 | 1278 | 1239 | 1215 | 1230 | 1081 | 1229 | 0.44 | -50 mg | 0.71 |
| **Beverage** |  |  |  |  |  |  |  |  |  |  |
| Calories (kcal) | 10660 | 319 | 364 | 311 | 285 | 303 | 218 | 0.24 | -102 kcal | 0.04 |
| Saturated fat (g) | 10289 | 5.2 | 6.4 | 5.4 | 3.8 | 4.0 | 1.4 | 0.22 | -3.8 g | 0.05 |
| Trans fat (g) | 10177 | 0.1 | 0.1 | 0.1 | 0.0 | 0.0 | 0.0 | 0.20 | 0.0 g | 0.29 |
| Unsaturated fat (g) | 10175 | 3.2 | 4.2 | 3.2 | 2.7 | 2.8 | 1.1 | 0.24 | -2.1 g | 0.01 |
| Sugar (g) | 10240 | 51.1 | 55.0 | 49.4 | 48.2 | 51.4 | 45.0 | 0.40 | -6.1 g | 0.33 |
| Non-sugar carbohydrates (g) | 10240 | 5.3 | 7.7 | 4.9 | 4.9 | 4.6 | 2.4 | 0.21 | -2.8 g | 0.04 |
| Protein (g) | **10660** | **6.4** | **6.3** | **5.7** | **5.1** | **5.0** | **3.3** | **<0.01** | **-3.1 g** | **<0.01** |
| Sodium (mg) | 10650 | 83 | 164 | 148 | 130 | 180 | 102 | 0.57 | 19 mg | 0.68 |

*Note.* Boldface indicates statistical significance at *p*<0.05. The n indicates total number of items introduced in all years for that category. All estimates are adjusted for restaurant type, whether the restaurant is a national chain, the year the restaurant began labeling their menus with calories, and whether the item is categorized as a kid’s item, shareable, regional or offered for a limited time.

^a^ Included all menu categories except toppings & ingredients.

^b^ Included all menu categories except beverages and toppings & ingredients
