# Supplementary material for: Calorie and nutrient trends in large U.S. chain restaurants, 2012-2018
Source: PLoS One. 2020 Feb 10;15(2):e0228891. doi: 10.1371/journal.pone.0228891 (PMC7010289; doi:10.1371/journal.pone.0228891)
Supplement: S9 Table — Excluding items from restaurants that did not label their menus with calories by 2018 (N = 338). (DOCX) [file pone.0228891.s010.docx]

**S9 Table.** Predicted mean per-item calories, saturated fat, trans fat, unsaturated fat, sugar, non-sugar carbohydrates, protein and sodium for newly introduced items in 2013-2018. Excluding items from restaurants that did not label their menus with calories by 2018 (N=338).

| **Menu Category** | ***n*** | **Means** | | | | | | ***p*-value for trend** | **2013-2018** | |
| --- | --- | --- | --- | --- | --- | --- | --- | --- | --- | --- |
|  |  | **New in 2013** | **New in 2014** | **New in 2015** | **New in 2016** | **New in 2017** | **New in 2018** |  | **Change** | **p-value** |
| **Overall^a^** |  |  |  |  |  |  |  |  |  |  |
| Calories (kcal) | **23616** | **467** | **489** | **456** | **469** | **432** | **353** | **0.04** | **-114 kcal** | **0.02** |
| Saturated fat (g) | 22635 | 7.9 | 8.5 | 8.0 | 7.7 | 7.7 | 4.7 | 0.08 | **-3.2 g** | **0.01** |
| Trans fat (g) | 21377 | 0.2 | 0.3 | 0.3 | 0.2 | 0.2 | 0.2 | 0.28 | 0.0 g | 0.52 |
| Unsaturated fat (g) | 21351 | 11.2 | 11.4 | 11.6 | 13.1 | 10.7 | 7.8 | 0.20 | -3.4 g | 0.12 |
| Sugar (g) | 21648 | 35.5 | 35.4 | 32.7 | 28.5 | 31.0 | 31.3 | 0.27 | -4.2 g | 0.31 |
| Non-sugar carbohydrates (g) | 21608 | 22.3 | 24.0 | 21.7 | 23.2 | 34.7 | 15.4 | 0.87 | -6.9 g | 0.09 |
| Protein (g) | 23155 | 16.1 | 16.5 | 15.7 | 17.6 | 15.0 | 12.4 | 0.15 | -3.7 g | 0.14 |
| Sodium (mg) | 23360 | 694 | 738 | 714 | 807 | 699 | 568 | 0.47 | -126 mg | 0.35 |
| **Food^b^** |  |  |  |  |  |  |  |  |  |  |
| Calories (kcal) | 11972 | 610 | 599 | 596 | 602 | 548 | 541 | 0.22 | -69 kcal | 0.23 |
| Saturated fat (g) | 11610 | 10.6 | 10.8 | 10.5 | 10.6 | 11.1 | 9.4 | 0.55 | -1.2 g | 0.32 |
| Trans fat (g) | 10512 | 0.3 | 0.4 | 0.4 | 0.3 | 0.4 | 0.4 | 0.69 | 0.0 g | 0.81 |
| Unsaturated fat (g) | 10495 | 19.6 | 19.0 | 19.8 | 20.8 | 17.9 | 18.2 | 0.60 | -1.4 g | 0.58 |
| Sugar (g) | **10626** | **19.3** | **17.1** | **15.2** | **14.9** | **13.9** | **12.3** | **0.03** | **-7.0 g** | **0.03** |
| Non-sugar carbohydrates (g) | 10613 | 38.8 | 36.7 | 40.4 | 38.8 | 69.4 | 37.3 | 0.48 | -1.5 g | 0.74 |
| Protein (g) | 11754 | 25.8 | 26.0 | 25.3 | 26.4 | 23.9 | 25.7 | 0.81 | -0.1 g | 0.97 |
| Sodium (mg) | 11800 | 1298 | 1258 | 1248 | 1287 | 1150 | 1267 | 0.68 | -31 mg | 0.84 |
| **Beverage** |  |  |  |  |  |  |  |  |  |  |
| Calories (kcal) | 11644 | 320 | 359 | 310 | 287 | 297 | 220 | 0.19 | **-100 kcal** | **0.03** |
| Saturated fat (g) | 11025 | 5.1 | 5.8 | 5.4 | 3.7 | 3.9 | 1.2 | 0.19 | **-3.9 g** | **0.04** |
| Trans fat (g) | 10865 | 0.1 | 0.1 | 0.1 | 0.1 | 0.0 | 0.0 | 0.16 | 0.0 g | 0.22 |
| Unsaturated fat (g) | 10856 | 3.2 | 3.9 | 3.2 | 2.6 | 2.7 | 1.0 | 0.21 | **-2.2 g** | **0.01** |
| Sugar (g) | 11022 | 50.7 | 53.3 | 51.2 | 47.8 | 48.7 | 41.2 | 0.25 | -9.5 g | 0.15 |
| Non-sugar carbohydrates (g) | 10995 | 5.3 | 8.7 | 5.0 | 5.4 | 4.7 | 2.7 | 0.12 | **-2.6 g** | **0.04** |
| Protein (g) | **11401** | **6.2** | **6.1** | **5.6** | **5.1** | **4.7** | **3.1** | **<0.01** | **-3.0 g** | **<0.01** |
| Sodium (mg) | 11560 | 81 | 162 | 148 | 133 | 171 | 107 | 0.61 | 26 mg | 0.57 |

*Note.* Boldface indicates statistical significance at *p*<0.05. The n indicates total number of items introduced in all years for that category. All estimates are adjusted for restaurant type, whether the restaurant is a national chain, the year the restaurant began labeling their menus with calories, and whether the item is categorized as a kid’s item, shareable, regional or offered for a limited time.

^a^ Included all menu categories except toppings & ingredients.

^b^ Included all menu categories except beverages and toppings & ingredients.
