# Supplementary material for: Calorie and nutrient trends in large U.S. chain restaurants, 2012-2018
Source: PLoS One. 2020 Feb 10;15(2):e0228891. doi: 10.1371/journal.pone.0228891 (PMC7010289; doi:10.1371/journal.pone.0228891)
Supplement: S10 Table — (DOCX) [file pone.0228891.s011.docx]

**S10 Table.** Predicted mean per-item calories, saturated fat, trans fat, unsaturated fat, sugar, non-sugar carbohydrates, protein and sodium for newly introduced items in 2013-2018 where all items >2000 calories (N=24) and all appetizers & sides >1000 calories (N=52) are flagged as shareable.

| **Menu Category** | ***n*** | **Means** | | | | | | ***p*-value for trend** | **2013-2018** | |
| --- | --- | --- | --- | --- | --- | --- | --- | --- | --- | --- |
|  |  | **New in 2013** | **New in 2014** | **New in 2015** | **New in 2016** | **New in 2017** | **New in 2018** |  | **Change** | **p-value** |
| **Overall^a^** |  |  |  |  |  |  |  |  |  |  |
| Calories (kcal) | **23954** | **479** | **486** | **455** | **456** | **428** | **357** | **0.02** | **-122 kcal** | **0.01** |
| Saturated fat (g) | 22961 | 8.2 | 8.5 | 8.0 | 7.5 | 7.6 | 4.8 | 0.06 | **-3.4 g** | **0.00** |
| Trans fat (g) | 21693 | 0.2 | 0.3 | 0.2 | 0.2 | 0.2 | 0.2 | 0.10 | -0.1 g | 0.20 |
| Unsaturated fat (g) | **21667** | **12.2** | **11.8** | **11.5** | **12.4** | **10.1** | **7.7** | **0.04** | **-4.5 g** | **0.02** |
| Sugar (g) | 21971 | 32.7 | 33.4 | 31.8 | 29.4 | 32.8 | 31.3 | 0.71 | -1.4 g | 0.70 |
| Non-sugar carbohydrates (g) | 21921 | 25.7 | 24.3 | 22.1 | 21.2 | 33.6 | 15.3 | 0.68 | -10.4 g | 0.02 |
| Protein (g) | **23471** | **17.0** | **16.8** | **15.9** | **16.9** | **14.3** | **12.7** | **0.02** | **-4.4 g** | **0.04** |
| Sodium (mg) | 23686 | 738 | 753 | 717 | 774 | 670 | 585 | 0.26 | -153 mg | 0.23 |
| **Food^b^** |  |  |  |  |  |  |  |  |  |  |
| Calories (kcal) | 12308 | 621 | 602 | 586 | 586 | 524 | 548 | 0.09 | -73 kcal | 0.14 |
| Saturated fat (g) | 11934 | 10.8 | 11.0 | 10.3 | 10.4 | 10.6 | 9.6 | 0.36 | -1.2 g | 0.24 |
| Trans fat (g) | 10826 | 0.4 | 0.5 | 0.4 | 0.3 | 0.3 | 0.4 | 0.36 | 0.0 g | 0.98 |
| Unsaturated fat (g) | 10809 | 20.6 | 19.4 | 19.1 | 19.9 | 16.6 | 18.3 | 0.23 | -2.3 g | 0.27 |
| Sugar (g) | **10947** | **19.1** | **17.2** | **14.7** | **14.8** | **13.1** | **12.4** | **0.01** | **-6.7 g** | **0.02** |
| Non-sugar carbohydrates (g) | 10924 | 42.4 | 38.6 | 39.5 | 37.2 | 64.8 | 37.6 | 0.63 | -4.8 g | 0.25 |
| Protein (g) | 12068 | 26.6 | 26.0 | 25.0 | 25.6 | 22.9 | 25.9 | 0.50 | -0.7 g | 0.80 |
| Sodium (mg) | 12124 | 1320 | 1259 | 1230 | 1252 | 1113 | 1273 | 0.49 | -47 mg | 0.74 |
| **Beverage** |  |  |  |  |  |  |  |  |  |  |
| Calories (kcal) | 11646 | 316 | 355 | 304 | 283 | 301 | 230 | 0.23 | -86 kcal | 0.05 |
| Saturated fat (g) | 11027 | 5.0 | 6.0 | 5.2 | 3.7 | 3.8 | 1.5 | 0.20 | -3.5 g | 0.05 |
| Trans fat (g) | 10867 | 0.1 | 0.1 | 0.1 | 0.0 | 0.0 | 0.0 | 0.18 | 0.0 g | 0.25 |
| Unsaturated fat (g) | 10858 | 3.1 | 4.0 | 3.1 | 2.7 | 2.7 | 1.2 | 0.23 | **-1.9 g** | **0.02** |
| Sugar (g) | 11024 | 48.8 | 52.6 | 48.4 | 46.8 | 49.4 | 45.8 | 0.57 | -3.0 g | 0.63 |
| Non-sugar carbohydrates (g) | 10997 | 5.5 | 8.2 | 5.1 | 5.0 | 5.1 | 2.6 | 0.16 | **-2.9 g** | **0.01** |
| Protein (g) | **11403** | **6.1** | **5.9** | **5.4** | **4.9** | **4.8** | **3.4** | **<0.01** | **-2.7 g** | **<0.01** |
| Sodium (mg) | 11562 | 79 | 160 | 144 | 130 | 173 | 112 | 0.53 | 33 mg | 0.45 |

*Note.* Boldface indicates statistical significance at *p*<0.05. The n indicates total number of items introduced in all years for that category. All estimates are adjusted for restaurant type, whether the restaurant is a national chain, the year the restaurant began labeling their menus with calories, and whether the item is categorized as a kid’s item, shareable, regional or offered for a limited time.

^a^ Included all menu categories except toppings & ingredients.

^b^ Included all menu categories except beverages and toppings & ingredients
